# Effects of Water Content and Temperature on Bulk Resistivity of Hybrid Cement/Carbon Nanofiber Composites

**DOI:** 10.3390/ma13132884

**Published:** 2020-06-27

**Authors:** Kamila Gawel, Mohammad Ali Taghipour Khadrbeik, Ruben Bjørge, Sigurd Wenner, Bartlomiej Gawel, Amir Ghaderi, Pierre Cerasi

**Affiliations:** 1 SINTEF Industry, P.O.Box 4760 Torgarden, 7465 Trondheim, Norway; MohammadAli.TaghipourKhadrbeik@sintef.no (M.A.T.K.); ruben.bjorge@sintef.no (R.B.); sigurd.wenner@sintef.no (S.W.); Amir.Ghaderi@sintef.no (A.G.); pierrerolf.cerasi@sintef.no (P.C.); 2 Department of Materials Science and Engineering, Norwegian University of Science and Technology, Sem Sælands vei 12, 7491 Trondheim, Norway; bartlomiej.gawel@ntnu.no

**Keywords:** cement paste, carbon nanofibers, conductive, bulk resistivity, water content, elevated temperatures

## Abstract

Cement nanocomposites with carbon nanofibers (CNFs) are electrically conductive and sensitive to mechanical loads. These features make them useful for sensing applications. The conductive and load sensing properties are well known to be dependent on carbon nanofiber content; however, much less is known about how the conductivity of hybrid cement–CNF depend on other parameters (e.g., water to cement ratio (w/c), water saturation of pore spaces and temperatures above ambient temperature). In this paper we fill-in these knowledge gaps by: (1) determining a relationship between the cement–CNF bulk resistivity and w/c ratio; (2) determining the effect of water present in the pores on bulk resistivity; (3) describing the resistivity changes upon temperature changes up to 180 °C. Our results show that the increase in the water to cement ratio results in increased bulk resistivity. The decrease in nanocomposite resistivity upon a stepwise temperature increase up to 180 °C was found to be related to free water release from cement pores and the dry materials were relatively insensitive to temperature changes. The re-saturation of pores with water was not reversible with respect to electrical resistivity. The results also suggest that the change in the type of electrical connection can lead to two orders of magnitude different bulk resistivity results for the same material. It is expected that the findings from this paper will contribute to application of cement–CNF-based sensors at temperatures higher than ambient temperature.

## 1. Introduction

Cement nanocomposites have recently gained much attention. Nanoparticles are added not only to engineer the mechanical properties [1,2,3,4] of the composites but also to render cement electrically conductive and responsive to mechanical load [5,6].

It has been shown that cement materials with well dispersed electrically conductive fillers, such as metal fibers, graphite powder, carbon nanofibers and carbon nanotubes, show conductive properties above a percolation threshold [7,8,9,10,11,12,13,14]. The percolation threshold is a critical concentration of the dispersed material above which the dispersed particles form a continuous network [15,16] so that it conducts electric charge. Due to their conductive properties, as well as sensitivity to stress (through the piezoelectric effect), the hybrid cement materials are considered as excellent sensors in areas such as the structural health monitoring of reinforced concrete structures [8,17] and traffic monitoring [18,19,20]. In these sensing applications the hybrid cement materials function as signal transducers that translate changes in mechanical load or material failure into the changes in electrical conductivity. The structural health monitoring of reinforced concrete structures relies on detecting and localizing failures in the concrete [21,22,23]. When a fracture starts to propagate in the material, the effective resistivity of the sensors increases as the conductive network gets interrupted [21]. In a traffic monitoring application, the strain sensitivity of hybrid cement materials is utilized. The strain sensitivity of conductive cements relies on changes in electrical resistivity upon the application of mechanical load. The physical mechanism underpinning this phenomenon is associated with the connectivity between the conductive particles. When a uniaxial compression is applied to the material with embedded electrically conductive fillers, the inter-particle distance in the filler decreases, and new conductive paths are created. The closer the conductive particles are and the more interparticle connections that are created, the larger an electrical current can be established leading to a decrease in resistivity of the material [24].

It has been well established that increasing CNF content leads to a decrease in the bulk resistivity of CNF-filled materials, with the highest drop within the percolation threshold concentration range [16,25]. More precisely, the bulk resistivity decreases most significantly in the concentration range at which nanofibers start forming a connected network. However, CNF concentration is not the only parameter that may affect the bulk resistivity values. Changes in other compositional parameters (e.g., water content or the concentration of other fillers and additives) may also affect the cement bulk resistivity at given CNF concentrations [25,26]. The results published so far on the effect of water on cement–CNF conductivity leads to contradictory conclusions. Some authors report an increase in conductivity upon the drying of cement–CNF samples [26]. According to those authors, water forms an isolation layer between fibers that disappears after drying, leading to increased conductivity. Others report a resistivity decrease upon immersion in aqueous solutions [27].

So far, conductive cement materials were intended for applications at atmospheric conditions and thus studied at ambient temperatures only. The hybrid materials can, however, also find applications as sensors at conditions where temperatures significantly exceed room temperature or even the boiling point of water (100 °C). Thus, it seems important to define how CNF/cement materials behave at elevated temperatures, and what parameters can be important for designing conductive cement-based sensors for high temperature applications. This is the main subject of this paper.

## 2. Materials and Methods

### 2.1. Preparation of Cement–CNF Materials and Resistivity Measurements

Pyrograf PR-19 XT-LHT nanofibers (NF) from Applied Sciences Inc. were used in this work. NF were heat-treated at temperatures of 1500 °C, which chemically carbonized the vapor-deposited carbon present on their surface. Such a heat treatment, according to the supplier, produces nanofibers providing the highest electrical conductivity in nanocomposites. PR-19 has an average diameter of about 150 nanometers and a length in the range of 50–200 microns. The surface area of the NFs is estimated to be 15–20 m^2^/g. Figure S1 in the Supporting Information, shows TEM images of the fibers extracted from cement samples.

Due to their hydrophobic nature, CNFs require the application of a dispersant to improve the homogeneity of CNFs in a cement slurry. Typically, polymers, surfactants, or a combination of the two is used to improve the dispersion of CNFs in the cement slurry. It has been previously shown that, sometimes, the combination of a polymer with the surfactant gives better CNF dispersion in cement materials than the application of a polymer or surfactant alone [28]. Thus in this work, two different dispersant systems were used: (1) MasterGlenium SKY 899 (BASF) superplasticizer polymer (SP) and (2) a combination of SP with sodium dodecyl sulfate surface active agent (SDS) in 1:1 weight ratio.

**Figure 1:**
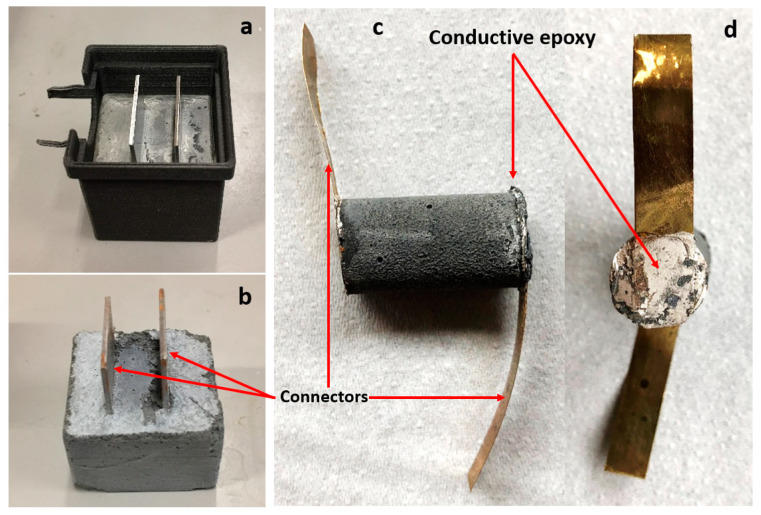
Photography of sample K in a 3D printed mold before (**a**) and after (**b**) removal from the mold, and sample C, side view (**c**) and top view (**d**), indicating metal connectors. Note, the connectors are embedded inside the hardened cement in samples K whereas the connectors are glued to the sides in samples C.

First, the CNF fibers were dispersed in the water/dispersant system. The CNF to dispersant weight ratio was 5:2. Next, the CNF dispersion was mixed with Portland G cement (Norcem) and additional water to yield the water to cement ratio, given in Table 1. Samples with the w/c ratio ranging between 0.49 and 0.66 were prepared. The CNF/cement weight ratio was constant and kept at 0.03 for all samples. The cement/CNF slurry was hand-mixed for 3 min and molded. Samples K1 and K2 were molded in 3D-printed cubic shaped forms with 3 cm long edges. Two metal plates separated by around 1 cm were placed in the middle and were acting as electrodes/connectors, as shown in Figure 1. presenting the K1 sample in the mold (a) and removed from the mold (b). Samples C1–C3 were molded in syringes with an internal diameter of around 12 mm. After one day of hardening at room conditions, the samples were placed in sealed plastic bags to prevent water evaporation. After two weeks of further hardening, the metal connectors were glued to the cylinder ends using a conductive (silver nanoparticles filled) epoxy resin (EpoTek, H21D), as shown in Figure 1c,d.

Resistance (R) between connectors was measured using a Fluke multimeter. The bulk resistivity (*ρ*, also called volume resistivity) of these materials were calculated according to Equation (1):

*ρ* = R*A/*l* (1)

where:

*R* is the electrical resistance measured between connectors,
*A* is the surface area of a connector,
*l* is the distance between connectors.

Resistance was measured for the samples at elevated temperatures. The Fluke 123 Scopemeter was used and was operating at a DC current of 0.5 mA or less (depending on the range), and an open voltage of no more than 4 V. The measurement was typically done in a heating oven approximately 30 min after the temperature of the heating oven was set at the test temperature and a relatively stable resistivity value was obtained.

The electrical resistance of cement materials without carbon nanofibers was in the range of tens of megaohms. The cement–CNF composite of the same geometry had electrical resistance in the kilohms range. The three orders of magnitude, or more, lower resistivity obtained for the composite samples suggests that the carbon nanofibers percolated in the cement matrix, giving rise to the electrical conductivity of composite materials.

### 2.2. X-Ray Micro-Computed Tomography (µ-CT)

X-ray micro-computed tomography (µ-CT) was performed on the cube samples in order to validate whether CNFs were homogeneously distributed using an industrial CT scanner (XT H 225 ST). It was operated at 210 kV and with a current of 155 μA. A tin filter was used. The raw CT data were reconstructed into cross-sectional slices. The resolution of the CT images was around 30 mm/1310 pixel = 0.02 mm/pixel. The material segmentation was done using Avizo Fire software.

### 2.3. Scanning Electron Microscopy (SEM)

Scanning electron microscopy (SEM) imaging was used to visualize the distribution of the CNFs within the cement matrix at a smaller scale. A Hitachi S3400N SEM was used for this purpose. The acceleration voltage was set to 10 kV and the images were acquired with a secondary electron detector.

### 2.4. Powder X-Ray Diffraction (XRD)

In order to quantify the amount of crystalline non-hydrated cement components, powder X-ray diffraction measurements were performed. Corundum (α-alumina) was used as an internal standard. The samples for XRD measurements were prepared by grinding the cement material together with corundum by hand using a mortar and pestle. The measurements were performed at room temperature, with the diffraction angle 2θ between 10° and 75° on a Bruker D8 Advance DaVinci diffractometer with Bragg–Brentano geometry, using CuKα radiation (λ  =  1.54187 Å). The X-ray powder diffraction pattern was collected over the course of one hour.

## 3. Results and Discussion

### 3.1. Significance of Electrical Connection Type for Bulk Resistivity of Cement–CNF Transducers at Higher Temperatures

**Figure 2:**
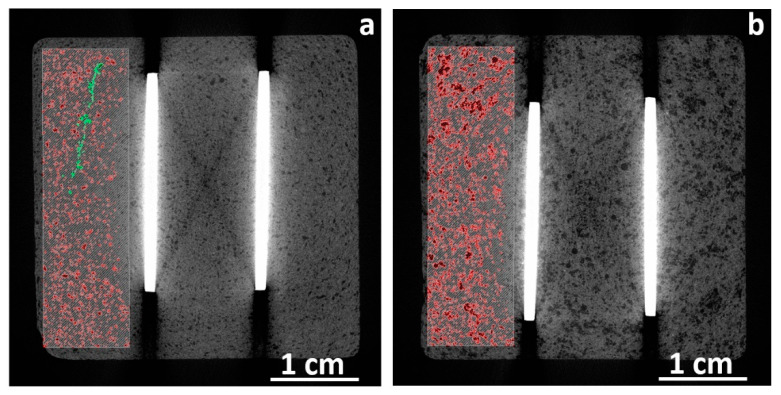
X-ray tomography cross-sections through samples K1 (**a**) and K2 (**b**). The red spots at the left side of the images indicate carbon nanofiber aggregates. The green pattern on figure (**a**) represents a fracture present in the material.

**Figure 3:**
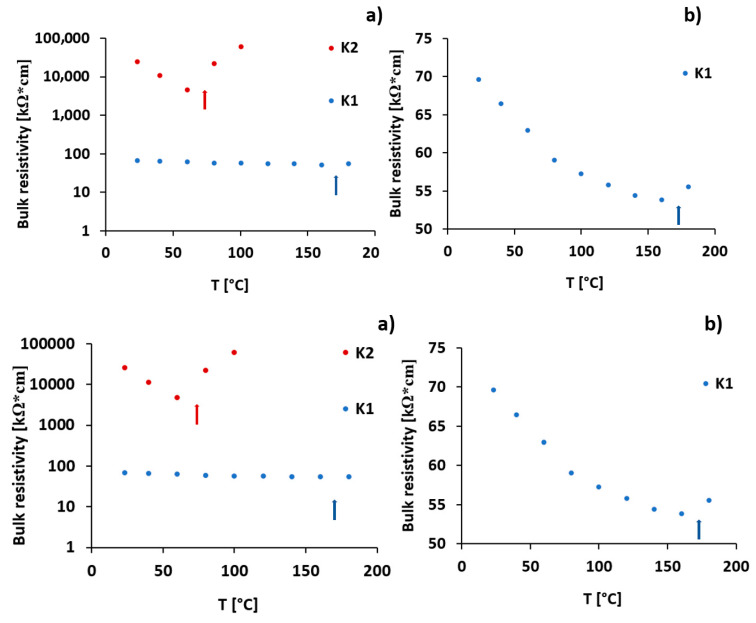
Bulk resistivity changes upon temperature increase for samples K1 and K2. (**a**) Logarithmic scale, and (**b**) linear scale concentrating on sample K1. Arrows show the temperature at which cracks propagated from electrodes towards the exterior.

K1 and K2 specimens were scanned using X-ray tomography (CT). The tomography cross-sections are presented in Figure 2. The brightest stripes in the middle of the figure are connectors made of steel that have high X-ray absorption coefficients compared to the cement material. The very dark strikes, visible on the pictures as an extension of very bright metal connectors, are scanning artefacts associated with beam hardening. The darker spots visible in sample K2, as shown in Figure 2b, within the grey-colored cement matrix, suggests that the CNF are inhomogeneously distributed within the cement. The presence of millimeter-sized darker spots suggests that CNFs, whose X-ray adsorption is lower compared to cement, are present in the form of large aggregates rather than well-distributed within the material. On the other hand, the 2D CT cross-sections through sample K1, as shown in Figure 2a, suggest that CNFs are well-distributed within the cement and much less aggregated at the scan resolution limit. Samples K1 and K2 contained identical weight fractions of CNFs; thus, it was expected that the CNFs in sample K1 formed better connected and thus perhaps better conducting networks. Indeed, the measured bulk resistivity at 23 °C for sample K1 was in the range of 70 kΩcm, while for sample K2 it was in the range of a few MΩcm, as shown in Figure 3.

**Figure 4:**
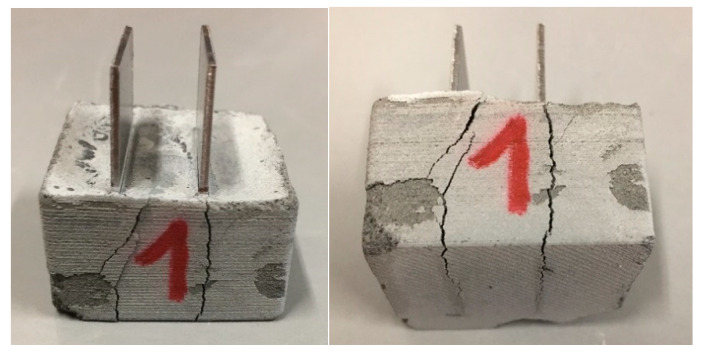
Photographs of specimen K1 with cracks propagating along the metal connector surfaces.

Figure 3 shows the bulk resistivity changes for samples K1 and K2 with temperature increases up to 180 °C. A gradual decrease in bulk resistivity was observed for both samples. The decrease continued until the temperature reached 60 and 160 °C for sample K2 and K1, respectively, and then started to increase. The bulk resistivity of sample K1 decreased from 70 kΩ·cm at 23 °C to 55 kΩ·cm at 160 °C. The sudden increase in resistivity for both samples coincided with audible crack initiation. The sudden increase in bulk resistivity was an indication of a loss of electrical conductivity, resulting from the interruption in the CNF network between the metal connectors. The most likely reason for cement samples fracturing was the thermal expansion of the metal connectors and the associated development of tensile stresses within the specimens. Figure 4 shows photographs of specimen K1 after the thermal cycling. Two cracks were seen, propagating along the metal connector surfaces. This suggests a loss of connectivity between the metal connector surfaces and the CNF network in the cement. The increase in resistivity upon the fracturing of the conductive cement materials has already been reported in the literature and its application to the structural health monitoring of reinforced concrete structures has been suggested [21,22]. Our results suggest that the choice of connector material, form, and size are important factors that have to be considered when designing high temperature sensors based on conductive cements. Sample K1 fractured at a significantly higher temperature compared to K2. This is most likely due to the higher tensile strength of the samples with well dispersed CNFs. It has already been shown that the mechanical properties of cements with well distributed CNFs can be significantly improved compared to pure cements [29].

**Figure 5:**
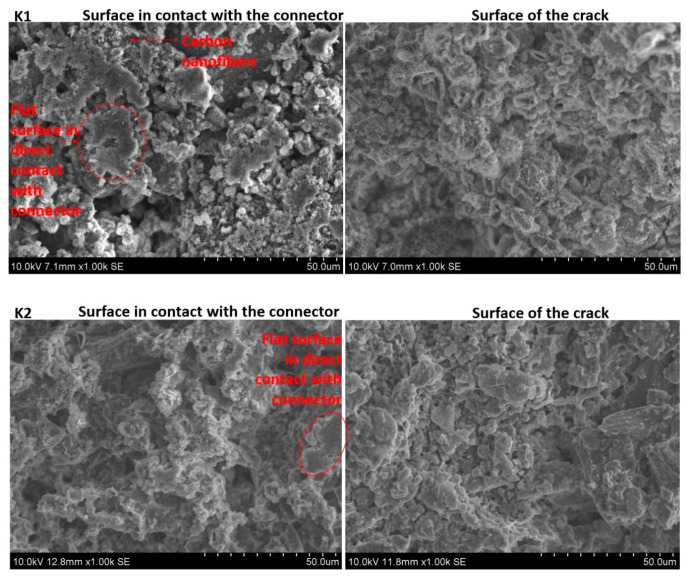
SEM (SE) images showing topography of K1 (**top**) and K2 (**bottom**) surface in contact with steel electrode (**left**) and surface of a fracture (**right**).

**Figure 6:**
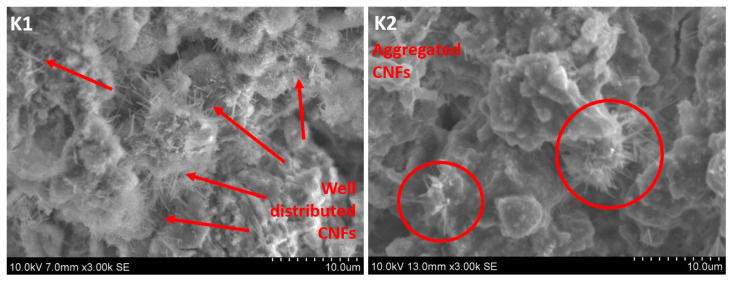
Typical higher magnification SEM (SE) images depicting homogeneous distribution of carbon nanofibers in sample K1 and aggregates of fibers in sample K2.

The surfaces of cement samples K1 and K2 that were in contact with the metal connector, as well as the surfaces of the fracture, were imaged using SEM. Typical images are presented in Figure 5. One striking difference in the appearance of the fracture surfaces between the samples is the presence of CNF fibers visible in sample K1 and their absence in sample K2. The fibers are evenly distributed over the fracture surface of sample K1. Given that the CNF contents are identical in samples K1 and K2, the lack of large numbers of CNFs at the crack surfaces of sample K2 should be attributed to its inhomogeneous distribution of CNFs. Indeed, higher magnification images of the two surfaces, as shown in Figure 6, indicate that the CNFs in sample K1 were homogeneously distributed, while in sample K2 they were present in the form of aggregates. This suggests that the superplasticizer polymer alone was a better dispersant of CNFs than the combination of polymer with a surface-active agent. It has been shown in the literature that, sometimes, a combination of polymer and surfactant or two surfactants can give better dispersion of CNFs than the surfactant or polymer on their own [28,30]. According to Wang Baomin [28], methyl cellulose polymer, when used together with sodium dodecyl sulfate, results in a more homogeneous dispersion of CNFs in cement than the polymer and sodium dodecyl sulfate alone. The differences in behavior between Wang’s system and the one described here is the nature of the polymer. Methyl cellulose is a nonionic polymer, while the superplasticizer polymer used in this work is most likely anionic as, according to the supplier, it is a modified polycarboxylate polymer. Although the exact structure of this polymer is not well described by the supplier, it can be expected that the polymer–surfactant interactions [31] in these two polymer–surfactant systems are significantly different, which is the most likely reason for the inconsistency in observations done by Wang and in this paper. On the other hand, some authors report a non-uniform distribution of CNFs in cement when using a polycarboxylate polymer only [32]. This may imply that the modified polycarboxylate superplasticizer polymer used in this work has better potential to disperse CNFs in cement than typical unmodified polycarboxylate superplasticizers. Another reason why the presence of sodium dodecyl sulfate (SDS) surfactant contributes to the larger inhomogeneity of the resulting composite material may be air entrapment. SDS, along with other surfactants, has shown suitability as air-entraining admixtures for cements [33]. Thus, it is likely that SDS had enhanced air entrapment in the composite, which led to the large material inhomogeneity, as well as higher electrical resistivity of the K2 composite.

**Figure 7:**
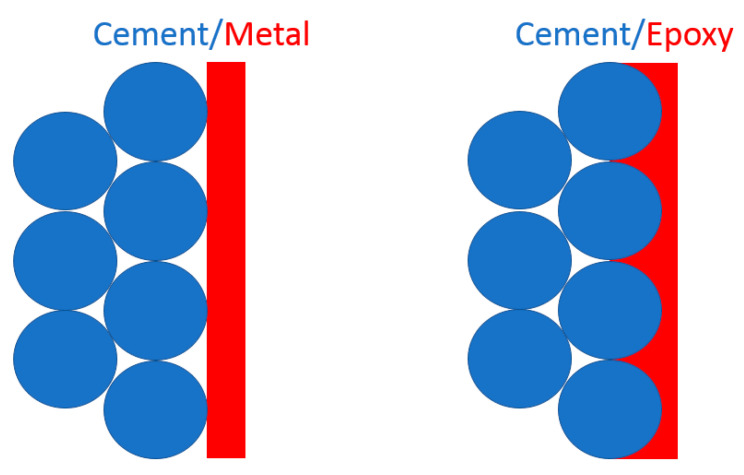
Schematic illustration of contact surface area between cement and electrical connector made of metal plate (**left**) and conductive epoxy resin (**right**).

It is obvious from the images taken from the surfaces in contact with the metal connector plates that only a small fraction of metal surface is in direct contact with the cement at the microlevel. This suggests that the calculations of bulk resistivity, that assume the whole surface of the connector being in direct touch with the cement, are burdened with a large error. This also suggests that the type of connection used in the sensor preparation has a large effect on the estimated resistivity values. For example, comparing bulk resistivity values for samples K1 and C2 at 23 °C can give an idea about how significant the effect of the connector type can be. Samples K1 and C2 have identical composition; the only difference is the incorporation of the metal connectors. Whereas in the case of sample K1 the connectors are embedded directly in the cement, sample C2 has connectors attached using a conductive epoxy resin (for details see the Materials and Methods section above). The bulk resistivity for K1 is calculated to be around 70 kΩ·cm, while for C2, the bulk resistivity value is almost two orders of magnitude lower and is around 850 Ω·cm. Given that the multiple reproducibility tests always resulted in the same order of magnitude values, the large difference in the bulk resistivity for samples K1 and C2 should be ascribed to differences in the incorporation of metal connectors. The conductive epoxy resin is filled with silver nanoparticles. Typically, epoxy resins are known for their good penetration into the pore structures of porous materials [34]. Thus, it is expected that the effective surface area of cement and CNFs, being in contact with conductive connectors, is higher for an epoxy connector than for a metal plate connector immersed in cement. This higher effective contact area between the conductive connector and the cement with the CNF network most likely contributes to the significantly lower bulk resistivity values observed in sample C2, compared to sample K1. The suggested mechanism underpinning this effect is illustrated in Figure 7. Summarizing, the contact resistance is important. The measured resistance is a net value of resistance for bulk material and the contact resistance between the cement composite and the electrical connector, as well as the contact resistance between the electrical connector and the multimeter probes. The contact resistance measured for the conductive epoxy itself was 0.2 Ω, so the multimeter and the conductive epoxy connection are regarded as conductive. If the contact resistance between the connector and the cement material is large, compared with the bulk resistance of the material, the contact resistance will be a limiting factor and, thus detecting small changes in the bulk resistance may be difficult.

### 3.2. Effect of w/c Ratio and Pore Water Content on Bulk Resistivity of Cement–CNF Materials

**Figure 8:**
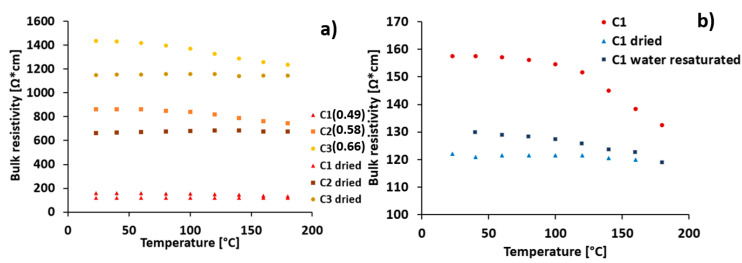
Resistivity changes upon temperature increase for samples with (**a**) different water to cement ratio: C1 (w/c: 0.49), C2 (w/c: 0.58), C3 (w/c: 0.66) and (**b**) different pore volume water saturation The resistivity changes for the reference sample without CNF is presented in Figure S2 in the Supporting Information.

Due to challenges associated with the fracturing of cement samples with embedded metal connectors upon temperature increase, the samples with epoxy-glued connectors were used to study the effect of temperature, water to cement ratio, as well as free water content on cement sensor resistivity. The bulk resistivity changes upon temperature increase up to 180 °C for the prepared samples, as well as the samples dried for one week at 40 °C, are presented in Figure 8. The results suggest that cement–CNF material bulk resistivity is strongly dependent on both the water to cement ratio and the amount of free water present in the cement pore system.

**Figure 9:**
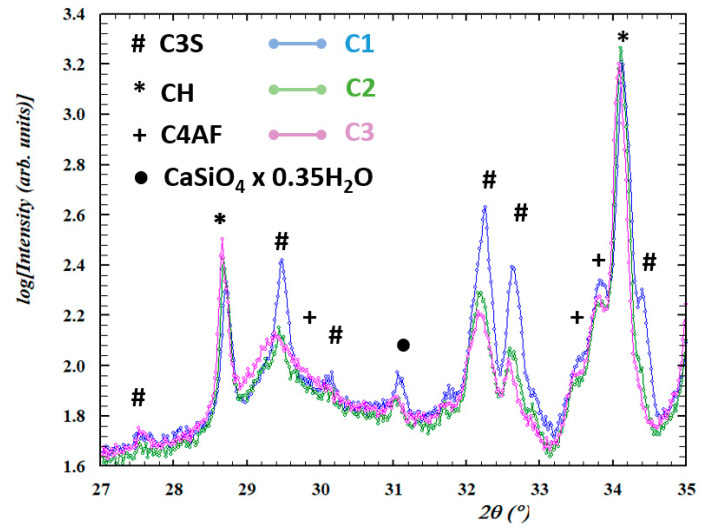
XRD diffraction patterns (normalized to corundum internal standard) for C1, C2 and C3 samples with indicated peaks from the most abundant crystalline phases present in the cement samples. C3S—tricalcium silicate (nonhydrated), CH—calcium hydroxide (main hydration product), C4AF—tetracalcium alumino ferrite.

**Figure 10:**
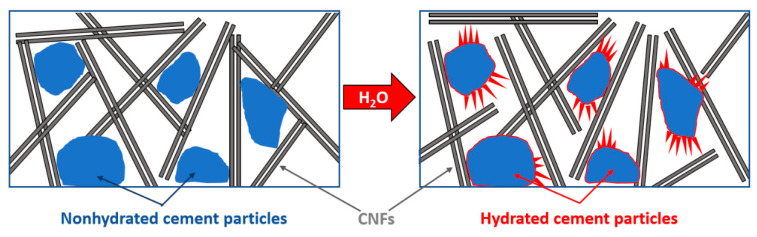
Schematic illustration of mechanism explaining increase in resistivity with increased water to cement ratio. The non-hydrated cement particles react with water. The hydration products (calcium silicate hydrate, calcium hydroxide) contribute to separation between conductive fibers.

The increase in the water to cement ratio at the stage of cement mixing results in increased bulk resistivity. The increased water to cement ratio typically coincides with the increased amount of hydration products. Indeed, quantitative analysis of XRD patterns, displayed in Figure 9, show increasing amounts of hydration products (calcium hydroxide, CH) and decreasing amount of non-hydrated substrates (tricalcium silicate hydrate, C3S) with increasing w/c content. Table 2 shows the contents of the most abundant crystalline phases present in the cement samples with respect to corundum as an internal standard. The higher the amount of hydration products, the more disturbed the network preformed at the stage of mixing and molding CNF. Figure 10 schematically presents how the hydration products may affect the CNF network. While mixing and molding, CNFs are distributed within non-hydrated cement particles and form the connected network. At the moment the cement powder is mixed with water, the hydration processes start. As a result of hydration, hydration products (calcium hydroxide, calcium silicate hydrate) are precipitated within the free spaces between the cement particles. This precipitation most likely contributes to increased separation between the CNFs in a twofold manner: (1) The distances between the CNFs may increase as a result of cement particle volume increase, associated with water binding and the precipitation of hydration products (amorphous calcium silicate hydrate and crystalline calcium hydroxide [35]) on their surface. (2) On the other hand, the nonconductive hydration products may precipitate in the spaces between fibers, leading to the loss of electric contact between them. The two effects may thus explain the increase in bulk resistivity upon w/c ratio increase.

The bulk resistivity of the prepared samples was sensitive to temperature changes, as shown in Figure 8. The decrease in bulk resistivity was observed upon a temperature increase from room temperature to 180 °C. However, the samples dried over a week at 40 °C were not significantly responsive to temperature increase. This suggests that the temperature sensitivity of the prepared samples is most likely due to water loss associated with the temperature increase. The dried samples had lower bulk resistivity values. This suggests that the presence of free water in the CNF–cement pore volume contributes to the electrical connectivity loss between CNFs. This is in line with the observations made by Zhang et al. [26] and Tzounis et al. [36], who hypothesize that water forms an insulating layer between fibers that is removed upon drying, which leads to an increase in conductivity. According to Sihai Wen et al. [37], in the dry cement–CNF composites, electronic conduction is dominating, while in the wet state, ionic conduction plays a significant role. In the wet state, highly concentrated electrolytes are present in the cement–CNF pore water [36], which contributes to conductivity. Nevertheless, the conductivity of our samples is higher after drying, and it can thus be concluded that the ionic conduction in the wet state is a less efficient conduction mechanism than electronic conduction in a dry state. Figure 8b compares resistivity changes as a function of temperature for the prepared, dried, and water re-saturated C1 sample. After re-saturation, the resistivity increases, but it does not reach the value measured for the original sample. The reason could be that the water, upon re-saturation, is unable to enter all the smallest micro- and mesopores from which it is removed upon drying. This could be due to the hydrophobic nature of the carbon nanofibers that, once aggregated, do not allow polar fluids to enter the spaces at their interface [38].

## 4. Conclusions

In this work the electrical response of hybrid, conductive cement–CNF materials to elevated temperatures has been studied. It has been shown that:

(1) The electrical response of these materials is related with two types of water present in the cement: (a) water that is mixed with cement powder and is partially consumed in cement hydration processes, and (b) free water that is present in cement pores that can be removed by drying.
(2) The increase in the water to cement ratio at the stage of cement mixing results in increased bulk resistivity. This has been attributed to the precipitation of larger amounts of hydration products that leads to a larger separation of carbon nanofibers and, thus larger resistivity.
(3) The material response to a stepwise temperature increase up to 180 °C is related to free water release from cement pores and the dry materials are relatively insensitive to temperature changes. The re-saturation of pores with water results in a slightly increased resistivity but the re-saturation process is not entirely reversible.
(4) The choice of electrical connector material, form, and size are important factors that have to be considered when designing high temperature sensors based on conductive cements. A very important design parameter that must also be taken into account is the effective contact surface area of the electrical connector material with the cement–CNF matrix. It has been shown that the change in the type of electrical connection can lead to two orders of magnitude different bulk resistivity results for the same material.

## Figures and Tables

**Table 1:** Sample overview indicating shape of the sample, dispersant type, weight ratios between carbon nanofibers (CNFs), water, dispersant and cement for each sample.

Sample Name	Form	Dispersant	CNF/Cement	Water/Cement	Dispersant/Cement
**K1**	cube	SP	0.03	0.58	0.012
**K2**	cube	SP + SDS	0.03	0.58	0.012
**C1**	cylinder	SP	0.03	0.49	0.012
**C2**	cylinder	SP	0.03	0.58	0.012
**C3**	cylinder	SP	0.03	0.66	0.012

**Table 2:** Weight content of the most abundant crystalline phases present in cement samples with respect to corundum as an internal standard.

	C1	C2	C3
**Corundum**	100.0	100.0	100.0
**CH**	8.1	9.3	10.0
**C4AF**	3.5	3.0	2.8
**C3S**	7.0	4.2	3.7

## Supplementary Materials

The following are available online at https://www.mdpi.com/1996-1944/13/13/2884/s1, Figure S1: TEM image of carbon nanofibers extracted from the cement sample K1 and K2, Figure S2: Resistivity changes upon temperature increase for samples with different water to cement ratio: C1(w/c = 0.49) C2 (w/c = 0.58) C3 (w/c = 0.66), and the reference sample (REF) without carbon nanofibers).

## References

[1] Golewski G.L. (2020). Energy Savings Associated with the Use of Fly Ash and Nanoadditives in the Cement Composition. Energies.

[2] Ahmed H., Bogas J.A., Guedes M. (2018). Mechanical Behavior and Transport Properties of Cementitious Composites Reinforced with Carbon Nanotubes. J. Mater. Civil Eng..

[3] Hawreen A., Bogas J.A. (2019). Creep, shrinkage and mechanical properties of concrete reinforced with different types of carbon nanotubes. Constr. Build. Mater..

[4] Hawreen A., Bogas J.A., Kurda R. (2019). Mechanical Characterization of Concrete Reinforced with Different Types of Carbon Nanotubes. Arab. J. Sci. Eng..

[5] Buasiri T., Habermehl-Cwirzen K., Krzeminski L., Cwirzen A. (2019). Piezoresistive load sensing and percolation phenomena in portland cement composite modified with in-situ synthesized carbon nanofibers. Nanomaterials.

[6] Zhou Z., Xie N., Cheng X., Feng L., Hou P., Huang S., Zhou Z. (2020). Electrical properties of low dosage carbon nanofiber/cement composite: Percolation behavior and polarization effect. Cem. Concr. Compos..

[7] Chung D. (2004). Electrically Conductive Cement-Based Materials. Adv. Cem. Res..

[8] Meoni A., D’Alessandro A., Downey A., García-Macías E., Rallini M., Materazzi A.L., Torre L., Laflamme S., Castro-Triguero R., Ubertini F. (2018). An Experimental Study on Static and Dynamic Strain Sensitivity of Embeddable Smart Concrete Sensors Doped with Carbon Nanotubes for SHM of Large Structures. Sensors (Basel, Switzerland).

[9] Carmona F., Canet R., Delhaes P. (1987). Piezoresistivity of heterogeneous solids. J. Appl. Phys..

[10] Wang X., Fu X., Chung D.D.L. (1999). Strain sensing using carbon fiber. J. Mater. Res..

[11] Sun M., Liu Q., Li Z., Hu Y. (2000). Study of piezoelectric properties of carbon fiber reinforced concrete and plain cement paste during dynamic loading. Cem. Concr. Res..

[12] Newnham R.E., Bowen L.J., Klicker K.A., Cross L.E. (1980). Composite piezoelectric transducers. Mater. Des..

[13] Li Z., Zhang D., Wu K. (2002). Cement-based 0-3 piezoelectric composites. J. Am. Ceram. Soc..

[14] Han B., Ou J. (2007). Embedded piezoresistive cement-based stress/strain sensor. Sens. Actuators A Phys..

[15] Dietrich Stauffer A.A. (1994). Introduction to Percolation Theory.

[16] Czyzewski J.B.P., Gaweł K., Meisner J. (2009). Rapid prototyping of electrically conductive components using 3D printing technology. J. Mater. Process. Technol..

[17] Ramam K., Chandramouli K. (2012). Piezoelectric cement composite for structural health monitoring. Adv. Cem. Res..

[18] Monteiro A.O., Loredo A., Costa P.M.F.J., Oeser M., Cachim P.B. (2017). A pressure-sensitive carbon black cement composite for traffic monitoring. Constr. Build. Mater..

[19] Zhang J., Lu Y., Lu Z., Liu C., Sun G., Li Z. (2015). A new smart traffic monitoring method using embedded cement-based piezoelectric sensors. Smart Mater. Struct..

[20] Wen S., Chung D.D.L. (2002). Piezoelectric cement-based materials with large coupling and voltage coefficients. Cem. Concr. Res..

[21] Saafi M. (2009). Wireless and embedded carbon nanotube networks for damage detection in concrete structures. Nanotechnology.

[22] Downey A., D’Alessandro A., Baquera M., García-Macías E., Rolfes D., Ubertini F., Laflamme S., Castro-Triguero R. (2017). Damage detection, localization and quantification in conductive smart concrete structures using a resistor mesh model. Eng. Struct..

[23] Baeza F.J., Galao O., Zornoza E., Garcés P. (2013). Multifunctional Cement Composites Strain and Damage Sensors Applied on Reinforced Concrete (RC) Structural Elements. Materials.

[24] Wen S., Chung D.D.L. (2006). Model of piezoresistivity in carbon fiber cement. Cem. Concr. Res..

[25] Yang N., Zhang K., Sun Q. (2018). Dispersion and Pressure Sensitivity of Carbon Nanofiber-Reinforced Polyurethane Cement. Appl. Sci..

[26] Zhang L., Ding S., Han B., Yu X., Ni Y.-Q. (2019). Effect of water content on the piezoresistive property of smart cement-based materials with carbon nanotube/nanocarbon black composite filler. Compos. Part A Appl. Sci. Manuf..

[27] Wang H., Shen J., Liu J., Lu S., He G. (2019). Influence of carbon nanofiber content and sodium chloride solution on the stability of resistance and the following self-sensing performance of carbon nanofiber cement paste. Case Stud. Constr. Mater..

[28] Wang Baomin F.C. (2020). Comparative Study on Dispersion of Carbon Nanofibers and Enhancement on Mechanical Property and Microstructure in Cement Composites. J. Nanosci. Nanotechnol..

[29] Yazdani N., Brown E., Loh K.J., Nagarajaiah S. (2016). 3-Carbon nanofibers in cement composites: Mechanical reinforcement. Innovative Developments of Advanced Multifunctional Nanocomposites in Civil and Structural Engineering.

[30] Luo J., Duan Z., Li H. (2009). The influence of surfactants on the processing of multi-walled carbon nanotubes in reinforced cement matrix composites. Phys. Status Solidi (a).

[31] Rosół K., Szczubiałka K., Jachimska B., Zapotoczny S., Nowakowska M. (2008). Interactions of a smart cationic polyelectrolyte based on hydroxypropylcellulose with an anionic surfactant. J. Appl. Polym. Sci..

[32] Yazdanbaksh A., Grasley Z., Tyson B., Abu Al-Rub R. (2009). Carbon Nanofibers and Nanotubes in Cementitious Materials: Some Issues on Dispersion and Interfacial Bond. ACI SP.

[33] Silva B.A., Ferreira Pinto A.P., Gomes A., Candeias A. (2020). Suitability of different surfactants as air-entraining admixtures for lime mortars. Constr. Build. Mater..

[34] Djouani F., Connan C., Delamar M., Chehimi M.M., Benzarti K. (2011). Cement paste–epoxy adhesive interactions. Constr. Build. Mater..

[35] Chavez Panduro E.A., Torsæter M., Gawel K., Bjørge R., Gibaud A., Bonnin A., Schlepütz C.M., Breiby D.W. (2019). Computed X-ray Tomography Study of Carbonate Precipitation in Large Portland Cement Pores. Cryst. Growth Des..

[36] Tzounis L., Liebscher M., Fuge R., Leonhardt A., Mechtcherine V. (2019). P- and n-type thermoelectric cement composites with CVD grown p- and n-doped carbon nanotubes: Demonstration of a structural thermoelectric generator. Energy Build..

[37] Wen S., Chung D.D.L. (2006). The role of electronic and ionic conduction in the electrical conductivity of carbon fiber reinforced cement. Carbon.

[38] Wandelt K. (2018). Encyclopedia of Interfacial Chemistry: Surface Science and Electrochemistry.

