# Supplementary material for: Effects of Water Content and Temperature on Bulk Resistivity of Hybrid Cement/Carbon Nanofiber Composites
Source: Materials (Basel). 2020 Jun 27;13(13):2884. doi: 10.3390/ma13132884 (PMC7372432; doi:10.3390/ma13132884)
Supplement: Supplementary file 1 [file materials-13-02884-s001.pdf]

materials

MDPI

Supporting information

Effects of Water Content and Temperature on Bulk Resistivity of Hybrid Cement/Carbon Nanofiber Composites

Kamila Gawel [1,*], Mohammad Ali Taghipour Khadrbeik [1], Ruben Bjørge [1], Sigurd Wenner [1], Bartlomiej Gawel [2], Amir Ghaderi [1] and Pierre Cerasi [1]

[1] SINTEF Industry, P.O.Box 4760 Torgarden, 7465 Trondheim, Norway; MohammadAli.TaghipourKhadrbeik@sintef.no (M.A.T.K.); ruben.bjorge@sintef.no (R.B.); sigurd.wenner@sintef.no (S.W.); Amir.Ghaderi@sintef.no (A.G.); pierrerolf.cerasi@sintef.no (P.C.)

[2] Department of Materials Science and Engineering, Norwegian University of Science and Technology, Sem Sælands vei 12, 7491 Trondheim, Norway; bartlomiej.gawel@ntnu.no

* Correspondence: kamila.gawel@sintef.no; Tel.: +47-9424-4083

Received: 15 May 2020; Accepted: 22 June 2020; Published: date

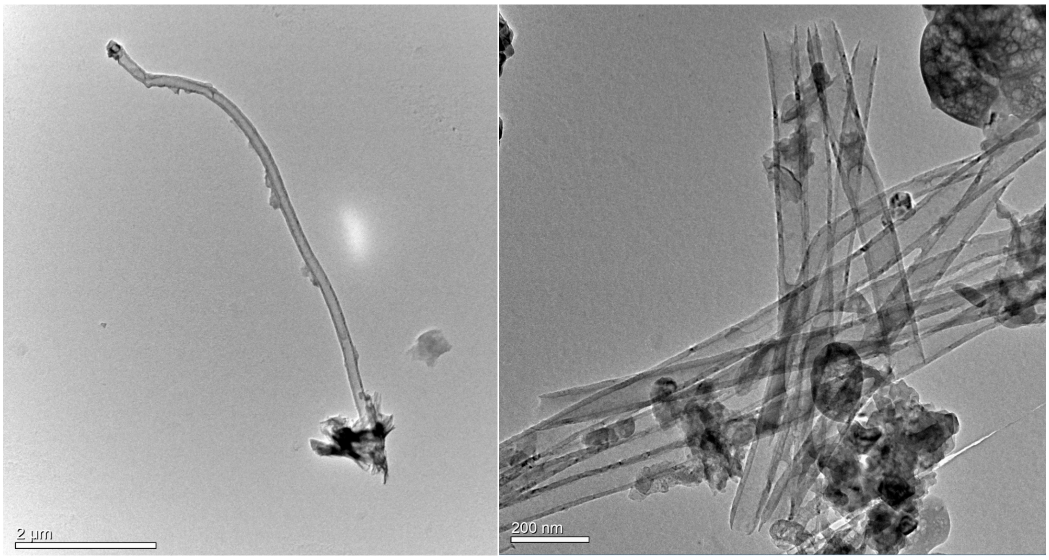

Figure S1. TEM image of carbon nanofibers extracted from the cement sample K1and K2.

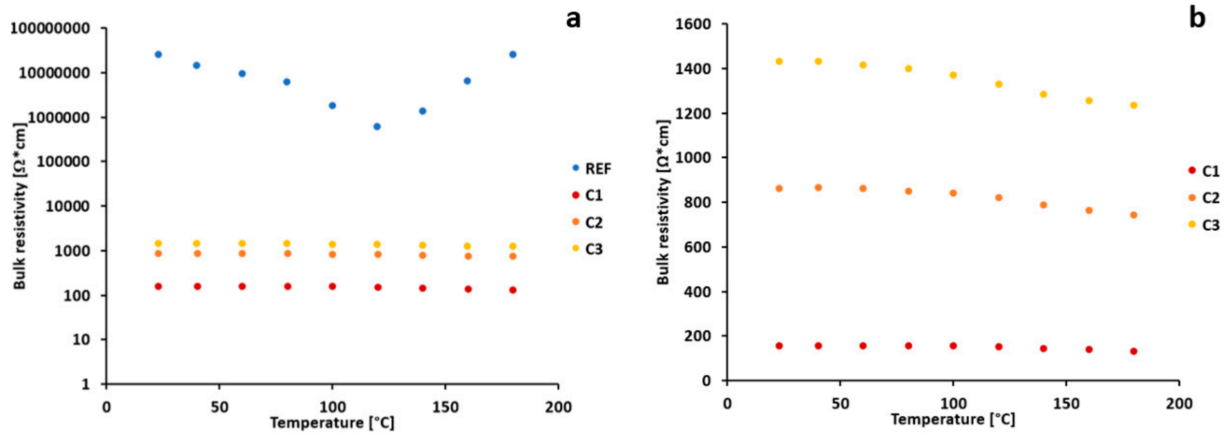

Figure S2. Resistivity changes upon temperature increase for samples with different water to cement ratio C1(w/c=0.49) C2 (w/c= 0.58) C3 (w/c= 0.66) and the reference sample (REF) without carbon nanofibers).

Due to very high resistivity values for the reference sample without CNFs the measured changes in bulk resistivity are not reliable.

© 2020 by the authors. Licensee MDPI, Basel, Switzerland. This article is an open access article distributed under the terms and conditions of the Creative Commons Attribution (CC BY) license (http://creativecommons.org/licenses/by/4.0/).
